# PI3Kδ Sustains Keratinocyte Hyperproliferation and Epithelial Inflammation: Implications for a Topically Druggable Target in Psoriasis

**DOI:** 10.3390/cells10102636

**Published:** 2021-10-02

**Authors:** Laura Mercurio, Martina Morelli, Claudia Scarponi, Giovanni Luca Scaglione, Sabatino Pallotta, Cristina Albanesi, Stefania Madonna

**Affiliations:** 1 Laboratory of Experimental Immunology, Istituto Dermopatico dell’Immacolata IDI-IRCCS, Via Monti di Creta, 104, 00167 Rome, Italy; martina.morelli@idi.it (M.M.); c.scarponi@idi.it (C.S.); g.scaglione@idi.it (G.L.S.); c.albanesi@idi.it (C.A.); s.madonna@idi.it (S.M.); 2 Integrated Center for Research in Psoriasis (CRI-PSO), Istituto Dermopatico dell’Immacolata IDI-IRCCS, Via Monti di Creta, 104, 00167 Rome, Italy; s.pallotta@idi.it

**Keywords:** PI3K isoforms, PI3Kδ, AKT, cytokines, psoriasis, hyperproliferation, seletalisib, skin inflammation, keratinocytes

## Abstract

The phosphatidylinositol 3-kinase (PI3K)-dependent signaling pathway is aberrantly activated in psoriatic lesions and contributes to disease pathogenesis. Among PI3Ks enzymes, PI3Kα, β, and δ isoforms are known to bind the p85 regulatory subunit and mediate activation of AKT and other downstream effectors. In this study, we deepened our understanding of the expression and function of PI3Kδ in skin lesions of patients affected by psoriasis. For the first time, we found that PI3Kδ is overexpressed in psoriatic plaques, and its expression is not only confined to infiltrating immune cells but also accumulates in proliferating keratinocytes of the epidermal basal layer. We investigated the function of PI3Kδ in psoriatic skin by evaluating the impact of seletalisib, a newly developed selective PI3Kδ inhibitor, in both in vitro and in vivo experimental models of psoriasis. Of note, we found that PI3Kδ sustains keratinocyte hyperproliferation and impaired terminal differentiation induced by IL-22, as well as induces epithelial inflammation and resistance to apoptosis mediated by TNF-α in human keratinocytes. Mechanistically, PI3Kδ promotes PDK1 phosphorylation and signals through AKT-dependent or -independent pathways. It is worth mentioning that PI3Kδ inhibition by seletalisib attenuates the severity of psoriasiform phenotype induced in the Imiquimod-induced mouse model of psoriasis by restoring the physiological proliferation and differentiation programs in epidermal keratinocytes and contrasting the cutaneous inflammatory responses. Therefore, we suggest PI3Kδ as a potential topically druggable target in psoriasis and skin diseases characterized by epidermal hyperproliferation and skin inflammation.

## 1. Introduction

Psoriasis is a chronic inflammatory skin disease characterized by epidermal alterations and a high number of skin-infiltrating immune cells [1,2]. In psoriatic skin, the inflammatory cell infiltrate includes polarized T-helper lymphocytes (Th)1; Th17- and Th22-releasing pro-inflammatory cytokines such as interleukin (IL)-17; IL-22; tumor necrosis factor α (TNF-α); and IFN-γ with a pathogenic action on epidermal keratinocytes. Indeed, these lymphocyte-released cytokines promote epidermal hyperproliferation and aberrant differentiation and induce the secretion of pro-inflammatory molecules, thus contributing to skin alterations and the manifestation of erythematous plaques [3,4,5,6].

In psoriasis, the inflammatory cytokines can elicit pathological processes via deregulation of several intracellular pathways. Among them, phosphatidylinositol 3-kinase (PI3K)-dependent signaling is reported to be aberrantly activated in psoriatic lesions and to contribute to disease pathogenesis [7,8,9,10,11]. Up-regulation of PI3K pathways has been reported also in the Imiquimod (IMQ)-induced mouse model of psoriasis [7,12,13], a disease system characterized by IL-23/IL-17-mediated responses [14]. Here, the modulation or inhibition of PI3K/AKT/mTOR signaling resulted in the amelioration of dermatitis, with reduction of epidermal hyperproliferation and skin inflammation [15,16]. 

PI3Ks are intracellular lipid kinases that phosphorylate the 3′-hydroxyl group of phosphatidylinositol and phosphoinositides [17]. This reaction leads to the activation of many intracellular signaling pathways that regulate cell metabolism, survival, and vesicle trafficking in mammalian cells. 

Among PI3K enzymes, class I PI3Ks consists of a catalytic p110 subunit (α, β, δ isoforms) that binds the p85 regulatory subunit (p85 α; β; or their splice variants p55α, p50α, or p55γ isoforms) and mediates activation of AKT and Mechanistic Target Of Rapamycin (mTOR) pathways [18,19]. PI3Kα (p110α) and PI3Kβ (p110β) subunits are ubiquitously expressed and display distinct roles in cellular signaling, cell growth, angiogenesis, and oncogenic transformation [20,21,22]. In contrast, PI3Kδ (p110δ) is mainly expressed by hematopoietic cells and is critical for full B- and T-cell antigen receptor signaling [16,23]. However, PI3Kδ expression has also been reported in non-leukocyte cell types, such as breast cancer cells [24], neurons [25], lung and synovial fibroblasts [26], and endothelial cells [27].

In human keratinocytes, Th1/Th17-released cytokines induce the activation of many PI3K downstream effectors, such as AKT, phosphoinositide-dependent kinase-1 (PDK1), and mTOR [7,8,28], which control the release of pro-inflammatory mediators [29]. Among them, AKT also enhances proliferation, impairs differentiation [30], and sustains anti-apoptotic processes in psoriatic keratinocytes [7]. Mechanistically, AKT protein needs to be phosphorylated in Ser473 and Thr308 to become fully active [31].

To date, the contribution of the PI3K isoforms, especially PI3Kδ, to epidermal hyperproliferation and skin inflammation in psoriasis skin has not been investigated. In this study, we deepened our knowledge of the expression of PI3K isoforms, and, in particular, the function of PI3Kδ in skin lesions of patients affected by plaque psoriasis. In contrast to isoforms α and β, we found that PI3Kδ is over-expressed in psoriatic skin lesions, and its expression is not only confined to infiltrating immune cells, as previously reported, but it also accumulates in the epidermis. In addition, we investigated the role of PI3Kδ in psoriasis skin by evaluating the impact of seletalisib, a newly-developed selective PI3Kδ inhibitor [32,33], in vitro, in keratinocytes activated by psoriasis-related cytokines and in vivo, in the IMQ-induced psoriasis-like model.

## 2. Materials and Methods

### 2.1. Geo Dataset

mRNA expression data were retrieved from two public NCBI Gene Expression Omnibus (GEO) databases, namely, GSE13355 and GSE41662 [34,35]. The same Affymetrix GPL570 (HG-U133_Plus_2, Affymetrix Human Genome U133 Plus 2.0 Array) platform was used in each study. Datasets were obtained from the transcriptome analysis of whole biopsies from lesional (LS) and non-lesional (NLS) psoriatic skin (*n* = 58 patients in the first study and *n* = 24 patients in the second one), compared to normal skin (*n* = 64 healthy controls from the first study).

### 2.2. Human Subjects

Skin biopsies were obtained from patients affected by plaque-type psoriasis (*n* = 6) afferent to the Dermatology Divisions of IDI-IRCCS and from healthy volunteers undergoing plastic surgery (*n* = 6). Biopsies were taken from the skin plaque at sites overlapping LS and NLS areas and were analyzed by immunohistochemistry [36,37]. Patients were enrolled in the study after giving their written and signed consent, with the approval of the IDI-IRCCS Local Ethics Committee (Prot. N. IDI-IMM-IL36pso) and according to the Declaration of Helsinki Guidelines.

### 2.3. Keratinocyte Cultures and Treatments

Human keratinocyte cultures were established from NLS skin of psoriatic patients and from skin of healthy subjects undergoing plastic surgery. Second- or third-passage cultured keratinocytes were used in all experiments, with cells cultured in the serum-free medium KGM (Clonetics, San Diego, CA, USA) for at least 3–5 days (about 70% confluence) before performing treatments with cytokines. Some experiments were performed on keratinocyte cultures undergoing terminal differentiation, achieved by growing cells at 100% of confluence (T0) and keeping them in culture for 4 days (T4) thereafter.

Stimulations with recombinant human (rh) IFN-γ (200 U/mL), TNF-α, IL-22, or IL-17A (50 ng/mL; R&D Systems, Minneapolis, MN, USA) were performed in keratinocyte basal medium (KBM, Clonetics). Seletalisib (UCB5857, MedChemExpress, Monmouth Junction, NJ, USA, IC50 = 12 nM), Ly294002 (Selleckchem, Huston, TX, USA, IC50 = 0.5 μM for PI3Kα, IC50 = 0.57 μM for PI3Kδ, IC50 = 0.97 μM for PI3Kβ), and MK2206 (Selleckchem, IC50 for AKT1 = 8 nM) were administered by pre-treating cultures for 1 h before adding cytokines. Optimal seletalisib concentration (1 μM) showed the highest AKT phosphorylation reduction and lowest cytotoxicity (Supplementary Figure S1). MK2206 and Ly294002 were used at 5 μM on keratinocyte cultures.

### 2.4. IMQ-Induced Psoriasiform-Like Model

Eight-week female BALB/cJ mice (Harlan Laboratories, San Pietro al Natisone, Italy), treated for 5 consecutive days with 5% (62.5 mg) IMQ (ALDARA cream, Meda AB, Solna, Sweden) received daily topical administration of seletalisib (1 mM in 50 μL volume) (*n* = 6) or control vehicle (1:5 *v/v* DMSO/EtOH) (*n* = 6), starting on day 0 of IMQ administration. In parallel, other two IMQ-treated groups received Ly294002 (5 mM) or MK2206 (5 mM). Four mice groups did not receive IMQ: one group received only control vehicle and was used as negative control (*n* = 2), the others received only seletalisib (*n* = 3), Ly294002 (*n* = 2), or MK2206 (*n* = 2). On day 6, full thickness skin biopsies of the treated area were collected with an eight-mm biopsy puncher. Skin was either snap-frozen in liquid N_2_ for total RNA preparation or fixed in neutral buffered formalin (Sigma-Aldrich, St. Louis, MO, USA) for histopathological analysis. All mouse procedures were carried out in accordance with institutional standard guidelines. The experimental design has been authorized by the Italian Health Minister (protocol No. 774/2018-PR).

### 2.5. Immunohistochemistry

Paraffin-embedded sections were obtained from biopsies of healthy or psoriatic skin, including LS and NLS zones of evolving plaques. Immunohistochemistry analyses were performed using primary antibodies (Abs) against PI3Kδ (#34050S) and PI3Kα (#4249S) (all purchased from Cell Signaling, Danvers, MA, USA). Secondary biotinylated mAb and staining kit (Vector Laboratories, Burlinagame, CA, USA) were used to develop PI3K immune reactivity.

Paraffin-embedded sections were also obtained from murine skin tissues and analyzed for epidermal and scale thickness, as well as cell infiltrate number. Average epidermal and scale thickness was quantified by a researcher blind to the experimental groups, who took five measurements per three sections for each mouse. Cells infiltrating dermis were also counted in three skin sections for each mouse. Immunohistochemistry analyses were carried out using primary Abs against PI3Kδ (#34050S), phosphorylated-AKT (Ser473#3787S; Thr-308#9275S) (all purchased from Cell Signaling), Ly6G (#550291, BD Biosciences, Franklin Lakes, NJ, USA), CD3 (#A0452, Dako, Glostruk, Denmark), Ki67 (#NCLk67P, Novocastra, Wetzlar, Germany), keratin 10 (K10) (#PRB-159P, Covance, Princeton, NJ, USA), CD11c (#MON3371, Monosan, Uden, Netherlands), and immunoreactivities developed with secondary biotinylated mAbs and staining kits (Vector Laboratories, Burlingame, CA, USA). All sections were counterstained with Mayer’s Hematoxylin and eosin, and positivity was evaluated in five adjacent fields at a 200X magnification. A semiquantitative, four-stage scoring system was applied, ranging from negative immunoreactivity (0) to strong immunoreactivity (4+) for KRT10 and PI3Kδ. For each skin specimen, two sections were analyzed, and positive cells were evaluated in five adjacent fields.

### 2.6. RNA Isolation and Real-Time Polymerase Chain Reaction (PCR)

Total RNA from skin biopsies was extracted using RNeasy Lipid Tissue Kit (Qiagen, Chatsworth, CA, USA) and from keratinocyte cultures by using the TRIzol reagent (Invitrogen, Carlsbad, CA, USA). mRNA was reverse-transcribed into cDNA by using SuperScript IV VILO master mix (Invitrogen) and analyzed by Quant Studio 5 real-time PCR machine (Thermo Fisher, Waltham, MS, USA) using SYBR Green or Taqman PCR reagents. The primer pairs used are listed in Table 1 (Applied Biosystems). *Il-1β* and *Cxcl15* mouse genes were analyzed by the TaqMan gene expression assay (assay ID: Mm00434228-m1; Mm04208136-m1, respectively). mRNA levels were normalized to *HPRT1* mRNA expression. The values obtained from triplicate experiments were averaged, and data presented as mean 2^−ΔΔCT ± SD.

### 2.7. Immunoblotting and Densitometry

Protein extract preparation and immunoblotting were performed accordingly to standard procedures [36,38]. The Abs used for the study were as follows: anti-PI3Kα (#4249S), anti-PI3Kβ (#3011S), anti-PI3Kδ (#34050S), anti-p-STAT3 (Tyr705#9131S), anti-p-AKT (T308#9275S and S473#9271S), anti-p-PDK1 (S241#3061S), anti-PDK1 (#3062S), anti-p-S6 (S235/236#2211), anti-S6 (#2317S), and anti-p-p65 (S276#3033S) (all from cell signaling); anti-K10 (#PRB-159P) and anti-Loricrin (PRB-145P) (both from Covance); anti-cyclin D1 (#sc-20044), anti-STAT3 (C-20#sc-482), and anti-β-actin (all from Santa Cruz Biotechnology, Santa Cruz, CA, USA); and anti-keratin 5 (K5) (#MA5-14473, Invitrogen). Filters were properly developed with anti-mouse, anti-goat, or anti-rabbit Ig Abs conjugated to HRP using the ECL-plus detection system (Amersham, Dubendorf, Switzerland), or, otherwise, the SuperSignal West Femto kit (Pierce, Rockford, IL, USA). Immunoblots were subjected to densitometry using the ChemiDoc MP Imaging System (Bio-Rad, Hercules, CA, USA) supported by the Molecular Analyst software (https://imagej.nih.gov/ij/, accessed on 20 July 2021). Band intensities were evaluated in three independent experiments and reported as means of densitometric intensity (D.I.) ± SD.

### 2.8. Cytotoxicity Test

Cytotoxicity towards different doses of seletalisib was tested by measuring the activity of lactate dehydrogenase (LDH) released from keratinocyte cultures, using Cytotoxicity Detection Kit Plus-LDH (Roche Diagnostics, Milan, Italy) and following the manufacturer’s instructions.

### 2.9. Proliferation Assay

Proliferation of keratinocytes was evaluated by using CyQuant Cell proliferation Kit (ThermoFisher Scientific), which measures total DNA content. Briefly, 0.5–1 × 10^4^ cells were grown for 24, 48, and 72 h in 96-well plates, in starvation medium and, after pre-incubation with different doses of seletalisib (0.1–1–10 μM) and stimulation with IL-22 (50 ng/mL), stained with CyQUANT dye, whose emission fluorescence was measured at 530 nm in EnSight multimode plate reader (Perkin Elmer, Waltham, MC, USA). Alternatively, 5–8 × 10^4^ keratinocytes were seeded in 12-well plates and, the day after, starved. After specific treatments, viable cells were evaluated by Trypan blue exclusion test.

### 2.10. Scratch Assay

Cultured keratinocytes were grown at 90% confluence, and cell monolayers were scratched using a sterile p-200 pipette, to create uniform cell-free zones. After a serum-free medium wash, the cells were pre-treated with 1 μM seletalisib for 1 h and then stimulated with IL-22 (50 ng/mL). Microscopy pictures were taken with a digital camera at different time-points following IL-22 treatment. The residual gap between migrating keratinocytes was measured with a computer-assisted image analysis system (Axiovision; Zeiss, Oberkochen, Germany) and expressed as percentage of the initial scratched area.

### 2.11. Apoptosis Analysis

Apoptosis of keratinocytes was evaluated using the FITC Annexin V/propidium iodide (PI) apoptosis detection kit (BD Biosciences, Milan, Italy). Viable, necrotic, and apoptotic were analyzed by Accuri C6 Flow cytometer (BD) equipped with Cell Quest software. The percentage of Annexin V^+^, PI^+^, and Annexin V/PI^+^ cell populations was evaluated in cultures of healthy and psoriatic keratinocytes left untreated or treated with TNF-α in presence or absence of seletalisib.

### 2.12. Statistical Analysis

Statistical analysis was performed by Student’s *t* test, Mann–Whitney *U*, or ANOVA one-way tests as specified in the figure legends. Tukey’s test as multiple comparison test was applied to data analyzed with ANOVA one-way test. All analyses were conducted using Prism v.5.0 (GraphPad Software, La Jolla, CA, USA).

Values were expressed as mean + S.D., and statistical significance was assumed at a *p* value of 0.05 or less.

## 3. Results

### 3.1. PI3Kδ Is Highly Expressed in Psoriatic Skin Lesions and Is Induced by Inflammatory Cytokines in Proliferating Keratinocytes

**Figure 1:**
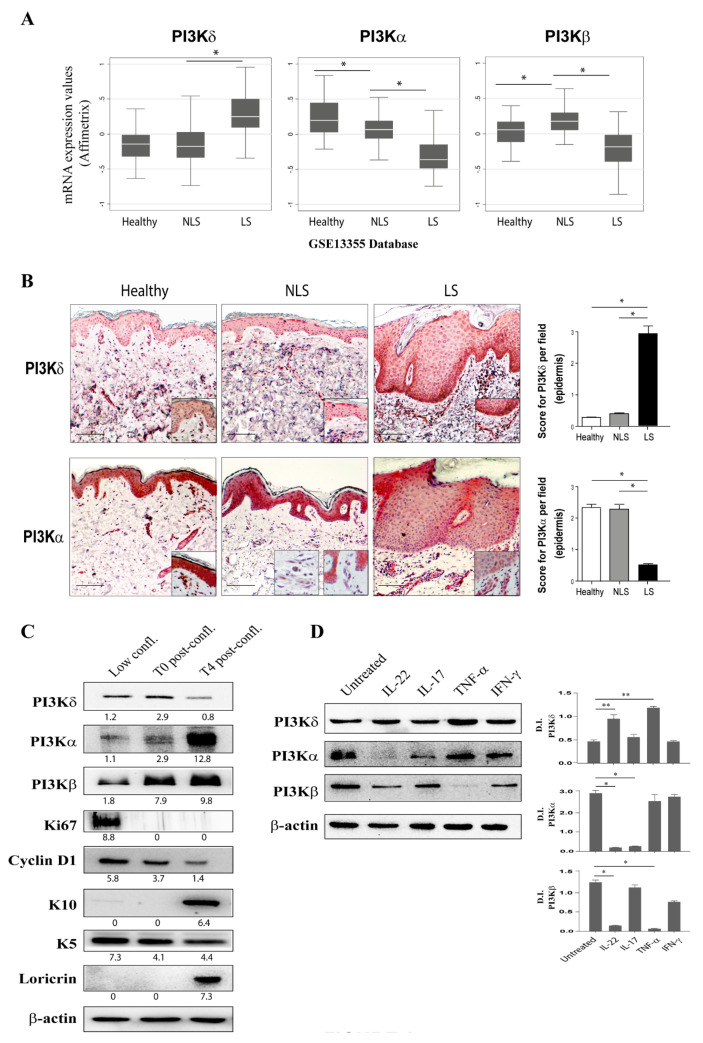
PI3Kδ expression is up-regulated in skin of psoriatic patients and in proliferating psoriatic keratinocytes activated by pro-inflammatory cytokines. (**A**) In GSE13355 dataset, the raw data from 180 microarrays were processed using the robust multichip average (RMA) method. The resulting expression values of the PI3Kδ, α, and β isoforms enzymes in healthy control (Healthy, *n* = 64), non-lesional (NLS, *n* = 58) and lesional (LS, *n* = 58) psoriatic skin tissues were obtained from RNA-seq dataset (GSE13355). Datasets were obtained from the transcriptome analysis of whole biopsies from lesional (LS) and non-lesional (NLS) psoriatic skin. Data are expressed as mean ± SD. Statistical significance was assessed by paired Student’s *t* test, * *p* ≤ 0.001. (**B**) Immunohistochemical (IHC) analyses for PI3Kδ and PI3Kα (stained in red) were performed on paraffin-embedded sections of biopsies obtained from psoriatic skin (*n* = 6), including NLS, lesional LS zones of evolving plaques, and healthy skin. Sections were counterstained with Mayer’s H&E. One out of six representative stainings of psoriatic skin biopsies are shown. Bars, 100 μm. All panels include 20X high magnifications panels. Graphs show the mean of four-stage score values ± SD for PI3Kδ or PI3Kα epidermal expression per three different fields of all six sections. * *p*  ≤  0.05, as assessed by Mann–Whitney *U* test. (**C,D**) Experiments were conducted on keratinocyte cultures isolated from psoriatic NLS skin (*n* = 3 strains) and maintained in different specific culture conditions. (**C**) WB analysis was performed on proliferating keratinocytes (sub-confluent), on confluent (T0) and post-confluent (T4) keratinocyte cultures, resembling different stages of differentiation in order to analyze the expression of PI3Kδ, -α, and -β isoforms; K10; Loricrin; K5; Ki67; and cyclin D1 proteins. β-actin was used as loading control. D.I. indicates the densitometric intensity of the indicated protein levels normalized to β-actin levels shown in one representative of three different WB. (**D**) The expression of PI3Kδ, -α, and -β isoforms was analyzed by WB on protein homogenates from proliferating keratinocytes left untreated or stimulated with IL-22, IL-17A, TNF-α, or IFN-γ for 6 h. β-actin was used as loading control. Graphs show the mean ± SD of densitometric intensity (D.I.) values of protein levels normalized to β-actin expression. Results were obtained by three independent experiments. * *p* ≤ 0.05, ** *p* ≤ 0.01 were assessed by paired Student’s *t* test comparing untreated and cytokine-treated cells.

In order to investigate on the expression of PI3K isoforms in skin of patients affected by psoriasis, two RNA-seq datasets (GSE13355 and GSE41662) relative to differentially expressed genes among healthy skin and diseased skin (asymptomatic NLS or LS skin) of patients with psoriasis were questioned. Interestingly, as shown in Figure 1A, we found that PI3Kδ was significantly upregulated in psoriatic LS skin compared to NLS and healthy skin biopsies. In contrast, PI3Kα mRNA levels were lower in LS biopsies compared to NLS skin, whereas PI3Kβ mRNA expression was significantly upregulated in NLS compared to healthy skin and diminished in LS group (Figure 1A).

To validate the expression of PI3K isoforms in psoriatic lesions and investigate on their localization in the skin cellular compartments, immunohistochemical analyses were performed on cutaneous biopsies from patients affected by plaque psoriasis, in particular in NLS, LS, and healthy tissues. Interestingly, as shown in Figure 1B, immunohistochemical analyses revealed that PI3Kδ was strongly expressed in epidermal compartment of LS biopsies, with an intense immunoreactivity in the basal layers of the epidermis where proliferating keratinocytes accumulate. Infiltrating immune cells were also positive for PI3Kδ in LS skin, in accordance with PI3Kδ expression previously reported in T lymphocytes and dendritic cells. In contrast, PI3Kδ seemed to be only slightly expressed in other resident skin cells, such as dermal endothelial cells (easily detectable around blood vessels), or in immune and skin-resident cells of NLS and healthy specimens. Furthermore, immunohistochemical analyses pointed out a significant reduction of PI3Kα isoform expression in epidermal keratinocytes of LS psoriatic skin in comparison to NLS and healthy skin, as well as a strong positivity in dermal endothelial cells detectable around blood vessels in all skin samples (Figure 1B). Unfortunately, the lack of specific antibodies against p110β isoform for immunohistochemical analysis, together with the low expression of p110β in skin, interfered with our attempts to examine the expression and localization of p110β isoform in psoriatic skin.

To better investigate the expression of PI3K catalytic isoforms in the epidermis of psoriatic skin, their protein levels were evaluated in proliferating (sub-confluent) and in post-confluent keratinocyte cultures (T4 post-confluent), with the latter resembling differentiated cells of the epidermal suprabasal layer. As shown in Figure 1C, PI3Kδ was expressed in proliferating keratinocytes, characterized by high levels of Ki67 and cyclin D1, two markers of cell-cycle progression and proliferation, respectively, and of K5, a marker of proliferating cells. PI3Kδ expression remained high in confluent (T0) cultures, but it declined in T4 post-confluent cultures, characterized by high levels of the keratinocyte differentiation markers loricrin and K10 (Figure 1C). Conversely, PI3Kα and PI3Kβ protein levels were low in proliferating cells and increased in keratinocytes undergoing differentiation (Figure 1C).

We further explored the expression of PI3K isoforms in response to IL-22, IL-17A, TNF-α, and IFN-γ, pro-inflammatory cytokines deeply involved in psoriasis pathogenesis. As shown in Figure 1D, PI3Kδ was significantly up-regulated by IL-22 and, to a greater extent, by TNF-α in psoriatic keratinocytes, as previously shown for human endothelial cells and synovial fibroblasts [27], whereas both PI3Kα and β isoforms were down-regulated by IL-22, with a complete abrogation of PI3Kα expression. In addition, TNF-α treatment strongly down-regulated PI3Kβ expression, without influencing PI3Kα levels, whereas IL-17A reduced the protein level of PI3Kα but not that of PI3Kβ (Figure 1D). Finally, IFN-γ weakly down-regulated PI3Kα and PI3Kβ expression, whereas slightly induced PI3Kδ.

Taken together, these data suggest a different involvement of PI3K isoforms in keratinocyte growth and differentiation. Of note, the upregulation of PI3Kδ by IL-22 and TNF-α in vitro, in keratinocyte cultures, as well as in vivo, in lesional psoriatic skin, suggests a role for PI3Kδ in keratinocyte proliferation and cutaneous inflammation.

### 3.2. Selective PI3Kδ Inhibition Interferes with PI3K-Dependent Pathways Activated by IL-22- and TNF-α in Psoriatic Keratinocytes

To assess the function of PI3Kδ in psoriasis skin, we evaluated the effects of a potent, ATP-competitive, and selective PI3Kδ inhibitor—seletalisib—on the key intracellular pathways known to be activated by IL-22 or TNF-α in human keratinocytes.

Previous in vitro binding and activity assays across a broad range of target classes showed that seletalisib is selective for PI3Kδ. From 239 kinases screened, seletalisib, at a concentration of 10 μM, showed no inhibitory activity greater than 47% (MAP4K4) against non-PI3K kinase enzymes and weak activities against non-kinase enzymes, such as phosphodiesterases. When screened at a concentration of 10 μM against 55 receptors and ion channels, the highest inhibitory activity of seletalisib observed was 20% [32].

Considering these biochemical data and our dose-response results on cytotoxicity and reduction of AKT and S6 phosphorylation in human keratinocytes, seletalisib was used at 1 μM optimal dose (Supplementary Figure S1). Thereafter, cultures of psoriatic keratinocytes were pre-treated with seletalisib for 1 h and then stimulated with IL-22 or TNF-α and analyzed at different time-points by Western blotting.

**Figure 2:**
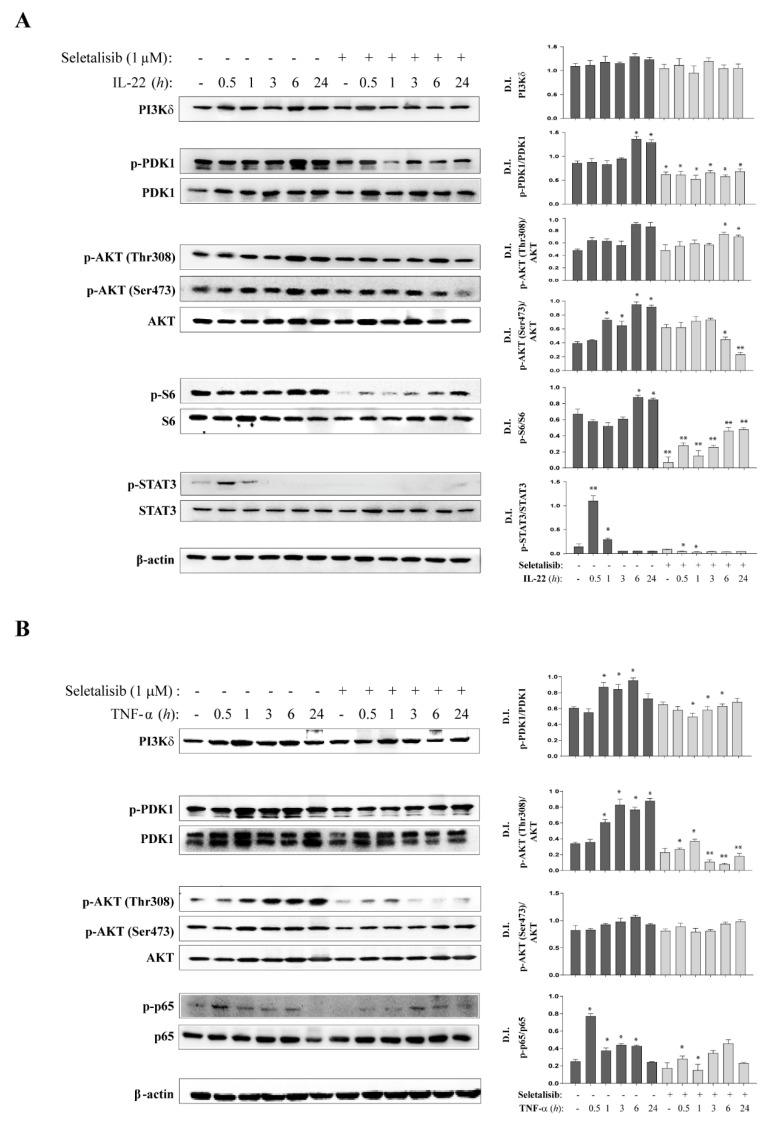
PI3K-dependent signaling pathways induced by IL-22 and TNF-α are down-regulated by selective PI3Kδ inhibition. (**A**,**B**) Protein extracts were obtained from psoriatic keratinocytes stimulated or not with IL-22 (**A**) or TNF-α (**B**) in presence or absence of seletalisib (1 μM) for the indicated time points and subjected to WB analysis to detect the expression of PI3Kδ the phosphorylation of PDK1 (S241) and AKT (Thr308, Ser473), as well as the phosphorylation of S6 in (**A**) and of p65 in (**B**). Filters were re-probed with anti-PDK1, -AKT (**A**,**B**), -S6 (**A**), and -p65 (**B**). β-actin levels were detected as a loading control. One representative experiment out of three performed is shown. (**A**,**B**) Graphs show mean of D.I. ± SD of bands obtained from WB analysis of protein extracts obtained from psoriatic keratinocytes (*n* = 3 strains). Data are expressed as mean ± SD of each D.I. value of total protein levels or the ratio of phosphorylated/unphosphorylated proteins normalized to β-actin expression. Results were obtained by three independent experiments. * *p* ≤ 0.05, ** *p* ≤ 0.01 were assessed by paired Student’s *t* test comparing resting with cytokine-treated cells, or seletalisib-treated with untreated cells.

PI3K cascade activation produces phosphatidylinositol (3,4,5) trisphosphates and leads to AKT activation by PDK-1 [39]. PI3Kδ activation by IL-22 was evident at 6 h and 24 h of stimulation concomitantly to the phosphorylation of PDK1. We found that, in accordance with previous studies [40], IL-22 induced a marked phosphorylation of AKT in Ser473 and a delayed phosphorylation in Thr308 residue. Consequently, S6, a downstream PI3K effector sustaining proliferation, was phosphorylated upon IL-22 stimulation, with the highest levels detected at 6 h and 24 h of stimulation (Figure 2A). As expected, seletalisib inhibited PI3Kδ and significantly reduced the phosphorylation of PDK1 during all time-points of IL-22 stimulation, whereas AKT phosphorylation in Thr308 and Ser473 was inhibited at 6 h and 24 h of stimulation. In contrast, seletalisib totally abrogated S6 phosphorylation, not only in IL-22-treated cells but also in untreated cells, suggesting a role for PI3Kδ in cellular homeostasis (Figure 2A). In addition, we found that activation of STAT3, the principal mediator of IL-22 signaling [41,42], was influenced by PI3Kδ activity. Indeed, seletalisib totally inhibited IL-22-induced STAT3 phosphorylation in Tyr705 at 30 min and 1 h of stimulation (Figure 2A), thus suggesting a possible molecular link between PI3Kδ and STAT3 in human keratinocytes, as previously demonstrated in PI3K-transformed cancer cells [43]. Finally, since seletalisib is an inhibitor of PI3Kδ enzymatic activity, we did not observe any significant change in PI3Kδ expression levels in psoriatic keratinocytes treated with seletalisib, compared to untreated cells upon IL-22 stimulation (Figure 2A).

Similarly, to IL-22, TNF-α induced the phosphorylation of PDK1 starting from 1 h of stimulation and upregulated AKT phosphorylation in Thr308, but not in Ser473 (Figure 2B). Consistently with previous studies, S6 phosphorylation was also strongly induced by TNF-α in keratinocyte cultures (data not shown). Importantly, the inhibition of PI3Kδ activity by seletalisib resulted in a significant reduction of phosphorylated PDK1 induced by TNF-a, and in a total abrogation of AKT phosphorylation from 1 h to 24 h of stimulation (Figure 2B). Contrarily, S6 phosphorylation was not influenced by seletalisib treatment (data not shown).

Finally, it has been previously reported that TNF-α-induced AKT activation determines the phosphorylation of the transcriptional factor p65 of NF-κB complex, thus leading to the translocation of p65 in the nucleus and activation of anti-apoptotic and inflammatory gene expression in human keratinocytes [7,44]. Consistently, we found that p65 phosphorylation was induced by TNF-α with a peak at 30 min, and it was inhibited by seletalisib treatment, with a significant effect at 30 min and 1 h of stimulation (Figure 2B). Additionally, upon TNF-α stimulation, seletalisib did not affect the expression of PI3Kδ itself in psoriatic keratinocytes (data not shown).

Taken together, these results suggest an active role for PI3Kδ in regulating the main intracellular pathways induced by pro-inflammatory cytokines in human keratinocytes.

### 3.3. PI3Kδ Sustains Proliferation, Migration, and Differentiation of Human Keratinocytes Regulated by IL-22

We further investigated the effects of PI3Kδ inhibition on the biological functions induced by psoriasis-related cytokines in human keratinocytes.

**Figure 3:**
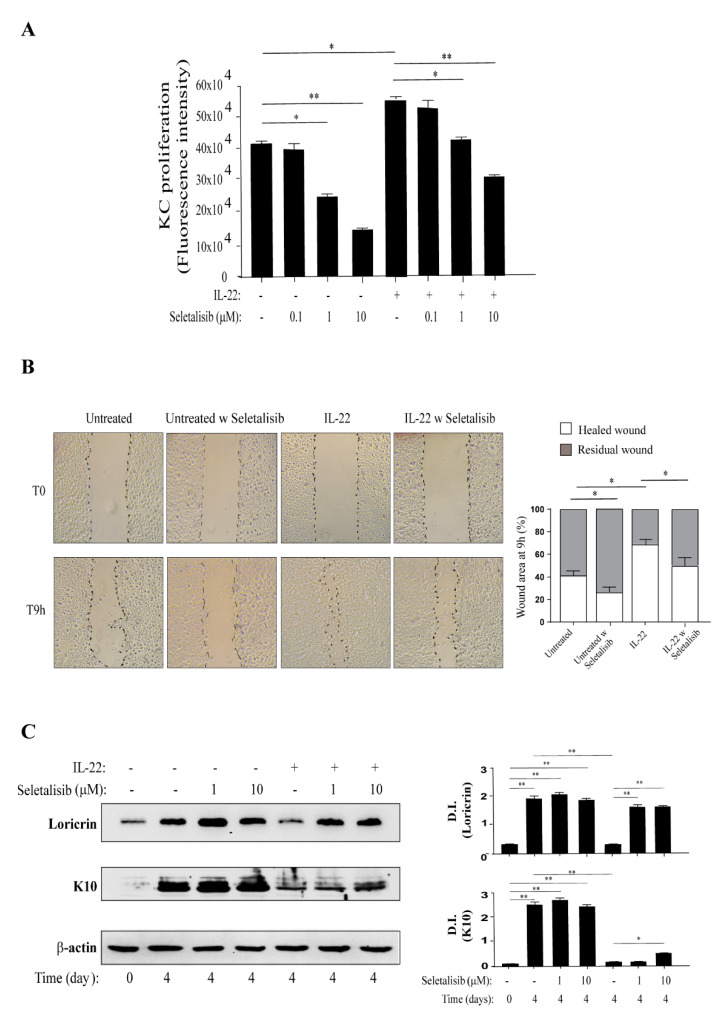
Selective PI3Kδ inhibition interferes with cell proliferation, migration, and differentiation, induced by IL-22 in psoriatic keratinocytes. (**A**) CyQUANT proliferation assay was performed to determine cell growth of keratinocytes left untreated or grown in presence of IL-22 for 72 h, pre-treated or not with different doses (from 0.1 to 10 μM) of seletalisb. Data are shown as mean values of fluorescence intensity obtained from three independent experiments ± SD. * *p* ≤ 0.05, ** *p* ≤ 0.01 calculated by one-way ANOVA test. (**B**) Scratch assays were carried out on keratinocytes left untreated or grown with IL-22, in presence or absence of seletalisib (1 μM) for 9 h. Microscopic images were taken immediately after (T0) and 9 h after wound induction on confluent cell layers (T9h). Initial scratches (0 h) were marked with black dashed lines. Cell-free area was measured and indicated as residual wound. Data are reported as healed wound (blank area of bars) vs. residual wound (grey area of bars). Data are shown as mean of percentage values obtained from three independent experiments ± SD. * *p* ≤ 0.05 was calculated by one-way ANOVA test. (**C**) Keratinocyte cultures were subjected to culture conditions determining terminal differentiation. The latter was achieved by growing cells at 100% of confluence (T0) and, thus, keeping them in culture for another 4 days in presence or absence of increasing seletalisib doses. Where indicated, cells were stimulated with IL-22. Loricrin and K10 protein levels were analyzed by WB, and one representative analysis is shown. Graphs show the mean of D.I. of the indicated proteins normalized for β-actin observed in three different WB. * *p* ≤ 0.05 and ** *p* ≤ 0.01 assessed by one-way ANOVA test. (**A**–**C**) Multiple comparisons were performed by Tukey’s test.

It has been established that IL-22 contributes to the aberrant proliferation and migration of keratinocytes, thus leading to the epidermis alterations commonly observed in psoriatic lesions [37,41,42,45,46,47]. In light of these assumptions, psoriatic keratinocytes were treated or not with increasing doses (0.1 to 10 μM) of seletalisib, stimulated with IL-22, and then analyzed in terms of proliferation and migration. As shown in Figure 3A, seletalisib counteracted proliferation induced by IL-22 in keratinocytes in a dose-dependent manner. Interestingly, consistently with the basal expression of PI3Kδ previously observed, pharmacological inhibition of PI3Kδ also reduced proliferation in untreated cells (Figure 3A) without interfering with their viability (Supplementary Figure S1), confirming a role of PI3Kδ in the proliferative status of epidermal keratinocytes. Furthermore, we analyzed the effects of seletalisib on the migration of psoriatic keratinocytes in an in vitro skin injury model, and we found that PI3Kδ inhibition significantly hindered the closure of wounds not only in IL-22-treated keratinocyte cultures but also in untreated cells (Figure 3B).

Other than inducing cell proliferation and migration, IL-22 interferes with keratinocyte terminal differentiation [42]. Thus, we analyzed the effects of different doses of seletalisib (1 or 10 μM) on cultures of psoriatic keratinocytes undergoing terminal differentiation upon IL-22 stimulation. As shown in Figure 3C, keratinocytes that underwent differentiation (4 days after 100% confluency) expressed higher levels of differentiation markers, such as loricrin and K10, as compared with proliferating cells (T0), whereas, as expected, IL-22 impaired their expression. Of note, seletalisib treatment restored the expression levels of loricrin at both doses, and slightly rescued K10 expression in keratinocyte cultures at the highest concentration (Figure 3C).

All these results suggest a crucial role for PI3Kδ activity in regulating the proliferative status and biological functions mediated by IL-22 in human keratinocytes.

### 3.4. PI3Kδ Chemical Inhibition Reduces the Expression of Inflammatory Genes and Increases Apoptosis in TNF-α-Activated Keratinocytes

**Figure 4:**
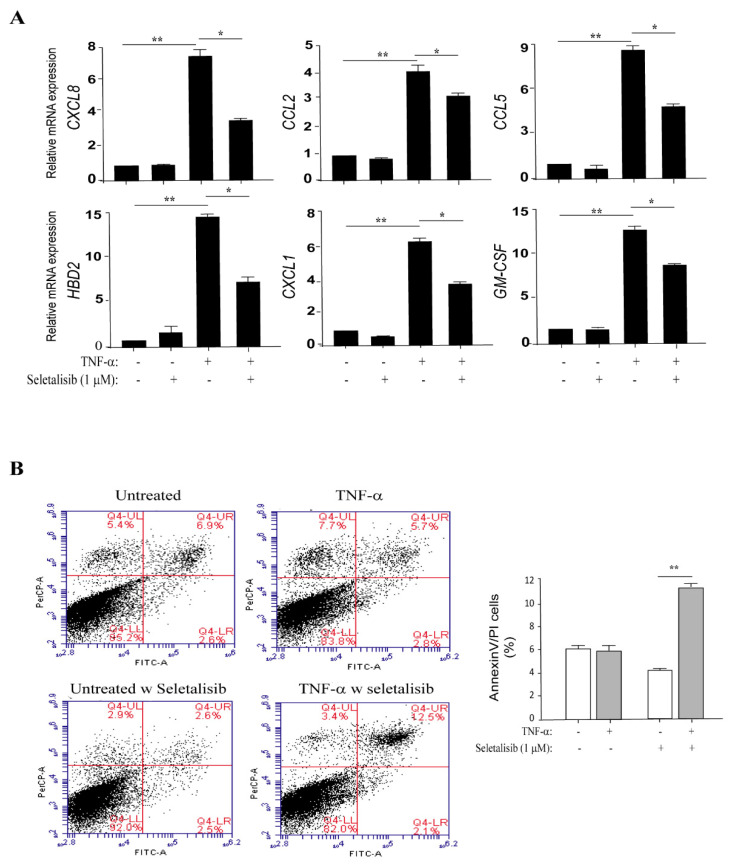
PI3Kδ inhibition reduces the expression of inflammatory genes and induces apoptosis in TNF-α-activated psoriatic keratinocytes. (**A**) Keratinocyte cultures were left untreated or stimulated with TNF-α in presence or absence of seletalisib (1 μM) for 8 h, and *CXCL8*, *CCL2*, *CCL5*, *HBD-2*, *CXCL1*, and *GM-CSF* mRNA expression was detected by real-time PCR and normalized to HPRT1 levels. Data shown are the mean of three different experiments ± SD. * *p* ≤ 0.05, ** *p* ≤ 0.01, as assessed by paired Student’s *t* test. (**B**) Psoriatic keratinocytes were pre-treated or not with seletalisb (1 μM) and then stimulated with TNF-α for 48 h. Apoptosis was evaluated by measuring Annexin/PI fluorescent staining through FACS analysis. Graphs show the mean ± SD of the percentage of AnnV/PI double-positive cells of three independent experiments. ** *p* ≤ 0.01, as assessed by paired Student’s *t* test.

We next evaluated whether PI3Kδ could influence the inflammatory responses of keratinocytes induced by TNF-α or IL-22 and mediated by PI3K-related pathways. To this end, the expression of a panel of molecules controlling or inducing skin inflammation was analyzed by real-time PCR in psoriatic keratinocyte cultures pre-treated with seletalisib and then stimulated with TNF-α or IL-22 for 18 h. As shown in Figure 4A, seletalisib significantly reduced the TNF-α-induced expression of *CXCL8*, *CCL2*, *CCL5*, *CXCL1*, *GM-CSF*, and the *HBD-2* antimicrobial peptide, whereas it did not affect the expression of *CCL20* chemokine and *IL-36γ*, *IL-6*, and *IL-1β* inflammatory cytokines (data not shown). Similarly, seletalisib could downregulate IL-22-induced expression of *CXCL1*, *CXCL8*, and *HBD-2* (Supplementary Figure S2). Minor inhibitory effects were observed in psoriatic keratinocytes stimulated with TNF-α and treated by Ly294002, a pharmacological inhibitor of all IA class PI3K isoforms (-α, -β, and -δ), or MK2206, a selective inhibitor of AKT1/2/3. In TNF-α-activated keratinocytes, the transcriptional expression of the only *HBD-2*, *CXCL8*, *CXCL10*, and *CCL2* genes was significantly reduced by MK2206 (73% for *HBD-2*, 49.5% for *CXCL8*; 54.3% for *CXCL10*; and 59.3% for *CCL2*, respectively, of expression reduction) or Ly294002 (90% for *HBD-2*, 88.2% for *CXCL8*; 72.3% for *CXCL10*; and 62.7% for *CCL2*, respectively, of expression reduction).

It is worth of note that psoriatic keratinocytes are characterized by a peculiar resistance to apoptosis that concurs to the thickening of psoriatic epidermis [48]. Multiple factors have been proposed to contribute to the reduced susceptibility of keratinocytes to apoptosis, including the enhanced levels of the anti-apoptotic molecules survivin and Bcl-xL, and hyperactivation of AKT, which can prevent cytokine-induced apoptosis via NF-κB pathway [7]. In this matter, we analyzed the effects of seletalisib on apoptosis of psoriatic keratinocytes following TNF-α stimulation by measuring Annexin (Ann V)/propidium iodide (PI) fluorescence. The same experiment was carried out on healthy keratinocyte strains, as control. As expected, TNF-*α* induced apoptosis in healthy keratinocytes, whereas it did not alter the apoptotic rate of psoriatic keratinocytes (Figure 4B). Interestingly, PI3Kδ inhibition by seletalisib rendered psoriatic keratinocytes more susceptible to TNF-*α*-induced apoptosis, with an increase of AnnV/PI positive cells in response to TNF-α treatment, whereas seletalisib treatment did not enhance the apoptotic rate in healthy strains (Supplementary Figure S3). These results can be explained with the sustained hyperactivation of AKT observed in the psoriatic keratinocytes analyzed in this study, in line with previous observations (data not shown).

In conclusion, all these data assess the involvement of PI3Kδ in sustaining the aberrant resistance to cytokine-induced apoptosis of psoriatic keratinocytes.

### 3.5. Topical Seletalisib Administration Ameliorates the Psoriasiform Phenotype of IMQ-Treated Mice

**Figure 5:**
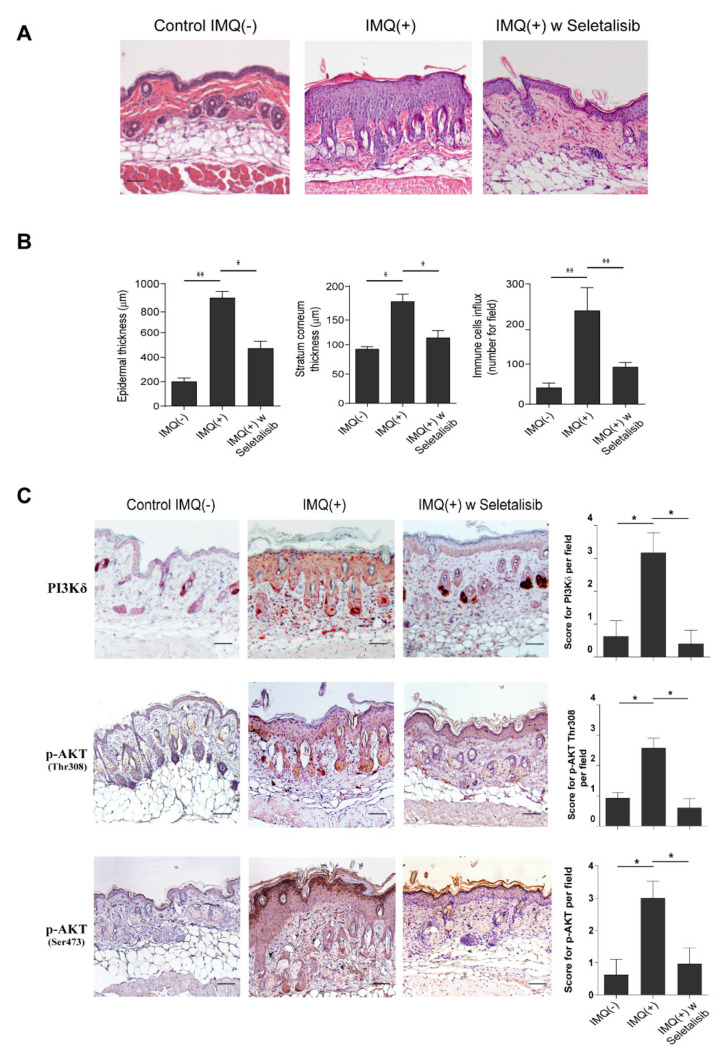
Ameliorative effects of seletalisib administration on pathological changes of IMQ-induced psoriasiform murine skin lesions. (**A**) Representative H&E staining of skin from Control IMQ (−), treated with IMQ cream (IMQ (+)), and IMQ-treated mice undergone to topical administration of seletalisib (IMQ (+) w seletalisib). Bars, 500 μm. (**B**) The quantifications of epidermal and stratum corneum thickness, as well as of immune cell influx, were analyzed as parameters of skin acanthosis and inflammation. Graphs show means of microns of epidermis and stratum corneum thickness, and mean of number of infiltrating immune cells per section (*n* = 3 sections) ± S.D. per group (*n* = 2 mice for Control IMQ (−); *n* = 6 mice for IMQ (+) and *n* = 6 for IMQ (+) w seletalisib groups). * *p*  ≤  0.05 and ** *p*  ≤  0.01, as assessed by unpaired Student‘s *t* test. (**C**) IHC staining of PI3Kδ isoform (red staining), p-AKT in Thr308, and in Ser473 (red-brown staining) was performed on skin of Control IMQ (−) (*n* = 2), IMQ (+) (*n* = 6), and IMQ (+) w seletalisib (*n* = 6). Sections were counterstained with Mayer’s H&E and were visually evaluated by a pathologist experienced in dermatology. One out of six representative stainings is shown. Bars, 500 μm. Graphs show the mean of four-stage score values for PI3Kδ and p-AKT (Thr308, Ser473) ± SD per three sections per all mice of each experimental group. * *p* ≤ 0.05, as assessed by unpaired Student‘s *t* test.

The impact of PI3Kδ inhibition was evaluated in vivo, in the experimental murine model of psoriasiform dermatitis induced by IMQ and characterized by the activation of IL-17/IL-23-dependent responses. Topical administration of seletalisib for 5 days, concomitantly to IMQ application, ameliorated the histological skin psoriasiform manifestations, with a reduction of ~50% of acanthosis and consequent scale thickness, and a substantial decrease of inflammatory cells infiltrating the dermis (~35% reduction) (Figure 5A,B). Stratum corneum thickness was also significantly reduced by seletalisib (~30% reduction). Similarly, Ly294002 or MK2206 were administrated daily in IMQ-treated murine groups, even though less pronounced effects on the infiltration of immune cells were observed when compared to seletalisib-treated group (Supplementary Figure S4A). However, the epidermal thickening was not affected by Ly294002, whereas it was only moderately reduced by MK2206 (Supplementary Figure S4A).

As expected, immunohistochemistry analysis showed that PI3Kδ was overexpressed in dorsal skin of IMQ-treated mice, and its expression localized not only in immune infiltrating cells but also in epidermal keratinocytes, with a high expression in proliferating cells of the basal layer (Figure 5C).

Consistent with a previous study [15], AKT phosphorylation in Thr308 was mainly detected in the nuclei of keratinocytes belonging to the of spinous and basal layers of the epidermis, with an expression pattern similar to PI3Kδ isoform expression profile (Figure 5C). In addition, AKT was strongly phosphorylated in Ser473 in keratinocytes of the granular upper layers of IMQ mice epidermis, with a prevalent cytoplasmic subcellular localization (Figure 5C).

It is worth is mentioning that the topical administration of seletalisib reduced the expression of PI3Kδ in both epidermal keratinocytes and infiltrating immune cells. Consequently, PI3Kδ inhibition resulted in reduced phosphorylation of AKT in both Thr308 and Ser473 sites (Figure 5C). In contrast, both Ly294002 and MK2206 treatments determined a weaker reduction of Ser473 phosphotylated AKT compared to seletalisib (Supplementary Figure S4B). Unfortunately, none of the antibodies tested in immunohistochemistry analysis permitted one to detect in vivo expression of phosphorylated PDK1 in IMQ model.

The impaired AKT phosphorylation in Thr308 and Ser473 determined by seletalisib was also confirmed by Western Blotting analyses carried out on protein homogenates of whole murine skin, as shown in Supplementary Figure S5. Furthermore, we found reduced levels of PI3Kδ in IMQ group treated by seletalisib, thus suggesting a feedback regulation of PI3Kδ on itself expression (Supplementary Figure S5). Consistently with immunohistochemical results, Western blotting analyses showed a hyperphosphorylation of PDK1 in IMQ mice compared to control, which was strongly reduced by PI3Kδ inhibition with seletalisib. In line with the pro-proliferative function of PI3Kδ, the reduced expression of PI3Kδ and downstream effectors was accompanied by a strong reduction of cyclin D1 expression, thus confirming a role for PI3Kδ in regulating keratinocyte proliferation (Supplementary Figure S5).

**Figure 6:**
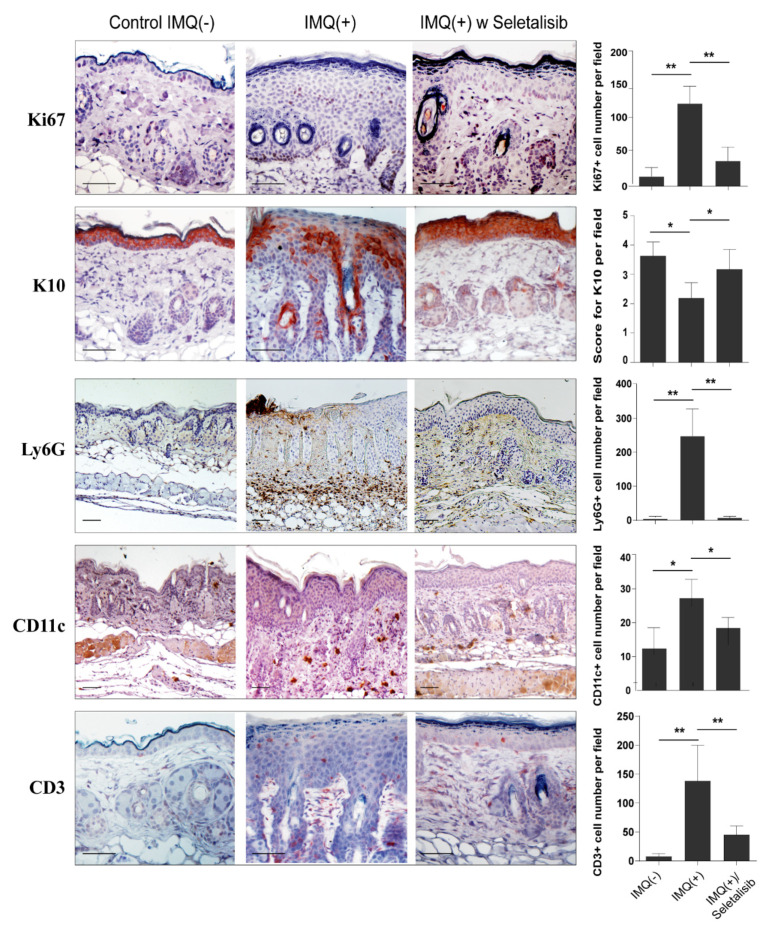
Seletalisib reverts the pathological markers in IMQ-induced murine model of psoriasis. In vivo expression of Ki67, K10, Ly6G, CD11c (all red-brown staining), and CD3 positve cells (red staining) was evaluated by IHC analysis on skin sample sections of control IMQ (−) (*n* = 2), IMQ (+) (*n* = 6), and IMQ (+) w seletalisib (*n* = 6) mice groups. Sections were counterstained with Mayer’s H&E and were visually evaluated by a pathologist experienced in dermatology. One out of ten representative stainings is shown. Bars, 200 μm. Graphs show the mean of number of positive cells for Ki67, Ly6G, CD11c, and CD3 expression analysis, or of four-stage score values for K10 ± SD per three sections per experimental group (*n* = 2 mice, Control IMQ (−); *n* = 6 mice, IMQ (+) and IMQ (+)/seletalisib groups). * *p* ≤ 0.05, ** *p* ≤ 0.01, as assessed by unpaired Student‘s *t* test.

To further deepen the effects of the pharmacological inhibition of PI3Kδ in IMQ-treated mice, we evaluated the expression of markers aberrantly observed in human psoriasis. As shown in Figure 6, seletalisib-treated group showed a reduced keratinocyte expression of the Ki67 proliferation marker as compared to IMQ group. In contrast, Ki67 in vivo expression was not affected neither by Ly294002 or MK2206 (Supplementary Figure S4B). Furthermore, PI3Kδ inhibition by seletalisib restored the expression levels of the differentiation marker K10, which is strongly diminished and delocalized in the epidermal compartment of IMQ-treated skin, and the typical compartmentalization to the upper granular layers observed in healthy skin (Figure 6). In addition, seletalisib strongly decreased the number of Ly6G^+^ neutrophils and infiltrating CD3^+^ T lymphocytes and moderately reduced the number of CD11c^+^ dendritic cells (Figure 6). The reduction of the number of Ly6G^+^ neutrophils was less significant in the skin of IMQ-treated mice who had undergone Ly294002 or MK2206 administration, whereas the decrease of the number of CD3^+^ T lymphocytes was similar in MK2206- and seletalisib-treated group (Supplementary Figure S4B). Notably, no changes were observed in murine skin treated by seletalisib, Ly294002, or MK2206 alone (data not shown).

**Figure 7:**
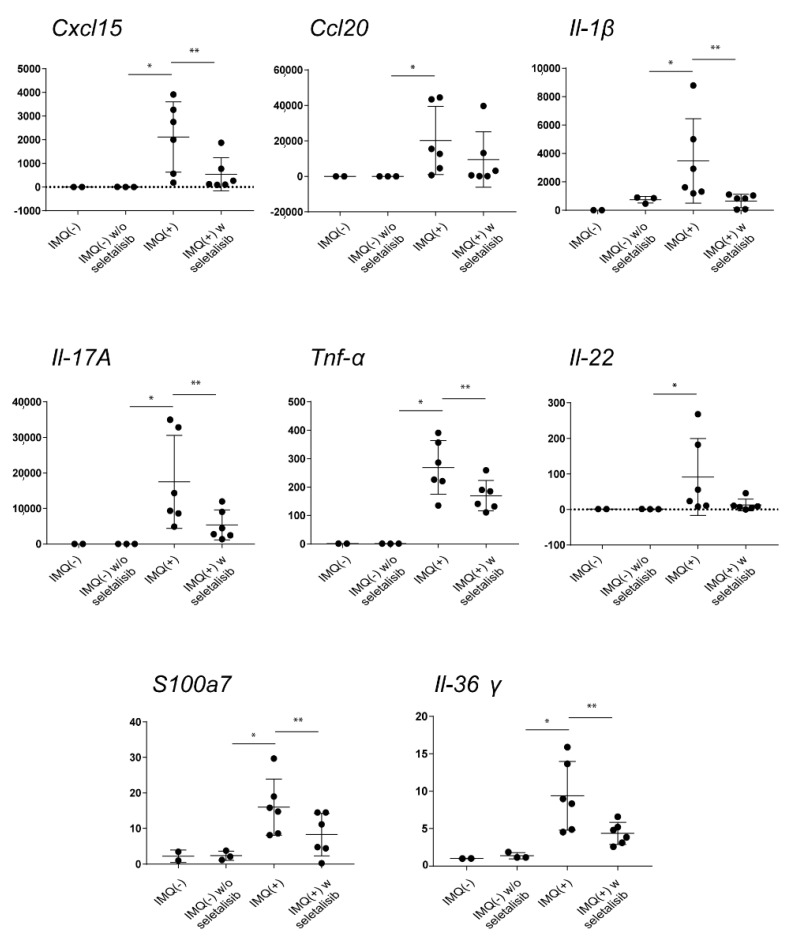
PI3Kδ inhibition by seletalisib counteracts the expression of pro-inflammatory molecules in IMQ-psoriasis-like mouse model. *Cxcl15*, *Ccl20*, *IL-1β*, *Il-17a*, *Tnf-α, Il-22, S100a7*, and *Il-36γ* mRNA expression was detected by real-time PCR performed on samples obtained from dorsal skin biopsies of mice treated as indicated (*n* = 2 mice, Control IMQ (−); *n* = 3 mice IMQ (−)/seletalisib group; *n* = 6 mice, IMQ (+) and *n* = 6, IMQ (+) w seletalisib groups). Results are shown as individual values of relative mRNA levels normalized to *Hprt1*, and include means ± SD, * *p* ≤ 0.05, ** *p* ≤ 0.01, as assessed by the Mann–Whitney U test.

Finally, we analyzed the effects of the pharmacological PI3Kδ inhibition on the mRNA expression of inflammatory mediators with a pathogenic role in IMQ-induced psoriasiform model, by performing transcriptional analysis on whole skin samples. As shown in Figure 7, a significant reduction of chemokine *Cxl15* and a slight decrease of *Ccl20* were detected in IMQ-induced mouse group treated by seletalisib (Figure 7). Seletalisib also reduced the expression of the pro-inflammatory cytokines *Il-1β*, *Il-17a*, *Tnf-α*, *Il-22*, and *Il-36γ*, and the anti-microbial peptide *S100a7*. All these molecules did not change in the groups treated by seletalisib alone (Figure 7).

The inhibitory effects of seletalisib on skin inflammation were broader than those determined by Ly294002 or MK2206. Indeed, Ly294002 reduced the expression of a limited number of chemokines and inflammatory cytokines in the dorsal skin of IMQ-induced psoriasiform murine model, and its inhibitory effects were less significant if compared to seletalisib (Table 2). MK2206 significantly reduced only *Cxcl15* mRNA expression (Table 2). Since the transcriptional analysis was performed on whole skin samples, we could not distinguish the anti-inflammatory effects of these inhibitors on epidermal compartment from those occurred on infiltrating immune or other resident dermal cells.

## 4. Discussion

Among class I PI3K enzymes, PI3Kδ expression and function have been widely investigated in immune cell populations. In a previous study, Roller and colleagues found that PI3Kδ expression is highly enriched in immune cells in IMQ-treated mice, and mice lacking functional PI3Kδ are largely protected from IMQ-induced psoriasiform dermatitis, correlating with reduced IL-17 levels in skin lesions, serum, and draining lymph nodes. Furthermore, in vitro studies demonstrated that PI3Kδ inhibitor IC87114 reduces IFN-γ production by human γδT cells, as well as IL-17 and IFN-γ production by PBMCs from psoriatic or healthy donors [15,16]. In addition, blocking of the PI3Kδ activity in T cells derived from patients affected by psoriasis counteracts their proliferation and activation [16].

In this study, we describe the expression and inflammatory function of PI3Kδ in psoriatic skin. To our knowledge, this is the first exploration on the role of this PI3K isoform in the pathogenic mechanisms executed by epidermal keratinocytes in psoriasis. We showed that PI3Kδ is overexpressed in skin lesions of psoriatic patients, and its expression is not only localized in immune cells infiltrating the dermis but also in epidermal keratinocytes, with a more pronounced expression levels in proliferating keratinocytes. Our in vitro studies confirmed the expression of PI3Kδ in human keratinocytes and its correlation with the proliferative status of cells, characterized by high levels of markers of cell-cycle progression and proliferation. Vice versa, PI3Kα and PI3Kβ isoforms are abundantly expressed in post-confluent differentiated keratinocytes, thus suggesting a role for PI3Kδ and PI3Kα/β in the switch from proliferation to differentiation of epidermal keratinocytes. RNA silencing experiments selectively targeting the three PI3K isoforms will permit one to better define their specific contribution to the keratinocyte maturation.

Among T lymphocyte-derived cytokines related to psoriasis, TNF-α is the main cytokine trigger of PI3Kδ expression, although IL-22 also sustains PI3Kδ levels in human keratinocytes, supporting a role for PI3Kδ in proliferation and de-differentiation processes induced by IL-22 in diseased skin. Consistently with PI3Kα expression observed in differentiated keratinocytes, IL-22 and IL-17A cytokines, both having de-differentiative functions, inhibited PI3Kδ expression, whereas PI3Kβ was strongly reduced by TNF-α. All these data explain the decrease of PI3Kα and PI3Kβ expression observed in psoriatic skin lesions, where epidermal keratinocytes are chronically exposed to inflammatory cytokines, such as IL-22, IL-17A, and TNF-α cytokines, and characterized by impaired differentiation.

Considering the enhanced expression of PI3Kδ in lesional psoriatic skin, we investigated the implication of PI3Kδ in disease pathogenesis by using a novel, potent, ATP-competitive, and selective inhibitor of PI3Kδ, known as seletalisib. Recent in vitro studies demonstrated that seletalisib interferes with proliferation and proinflammatory cytokines production in activated T lymphocytes [49,50]. Of note, seletalisib (UCB5857) has been orally administrated to patients with mild-to-moderate psoriasis in a phase-I clinical trial study, showing ameliorative effects on size and appearance of psoriatic lesions, together with reduction in T-cell and neutrophil skin infiltration [33]. However, the molecular and biological effects of PI3Kδ inhibition on resident skin cells, and in particular on epidermal keratinocytes, have not yet been investigated.

**Figure 8:**
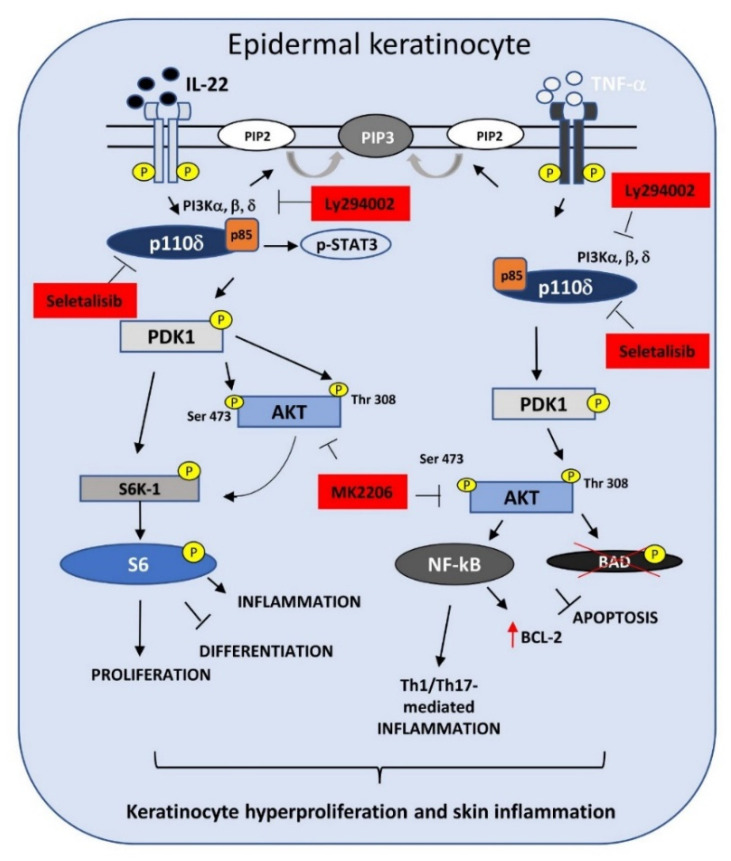
A graphical model on the role of PI3Kδ in regulating the molecular mechanisms and biological processes induced by TNF-α and IL-22 in human keratinocytes. PI3Kδ pathway can be activated upon stimulation of receptor tyrosine kinases (RTK) by TNF-α or IL-22 cytokines, leading to PI3Kδ activation which in turn phosphorylates PIP2 to PIP3. We assume that PI3Kδ activation by IL-22 leads to phosphorylation of PDK1, which results in activation of S6 ribosomal protein by the direct phosphorylation of S6K1 kinase or by the recruitment and activation of AKT. In addition, we hypothesize also a direct link between PI3Kδ and STAT3 molecule, independently from PDK1/AKT axis. These molecular events promote proliferation, inhibit terminal differentiation of epidermal keratinocytes, and sustain epithelial inflammation. In parallel, following the activation by TNF-α, PI3Kδ induces the phosphorylation of PDK1 and AKT, which in turn activates the transcriptional factor p65 of NF-κB complex, responsible for the expression of anti-apoptotic proteins, such as BCL-2. In parallel, the activation of AKT pathway leads to the phosphorylation and inactivation of the pro-apoptotic BAD molecule, and consequently to the suppression of pro-apoptotic mechanisms. Finally, the activation of NF-κB pathway induces the expression of a variety of inflammatory genes in human keratinocytes, contributing to the epithelial inflammation. In the model, the pharmacological inhibitors blocking their specific targets are also indicated in red.

Therefore, we evaluated the impact of PI3Kδ inhibition by seletalisib in experimental models of psoriasis, in particular in vitro, in keratinocytes activated by psoriasis-related cytokines, and in vivo, in a murine model of psoriasiform dermatitis induced by IMQ. Here, we propose a model in which PI3Kδ plays a central role in the molecular pathways and biological processes mediated by IL-22 and TNF-α in psoriatic skin (Figure 8). In support of this model, we provide evidence that PI3Kδ sustains the hyperproliferative, migratory, and de-differentiative action of IL-22 in human keratinocytes. However, we found that PI3Kδ also supports the physiological proliferation and migration of epidermal keratinocytes in resting conditions.

At molecular level, PI3Kδ mediates the IL-22-induced phosphorylation of the intracellular effector PDK1 and downstream AKT and S6 proteins. These results are in line with previous studies, demonstrating that PDK1 activates the intracellular AKT/S6K1/S6 axis in epithelial cell lines, breast cancer, and melanoma cells, thus controlling their proliferation and migration [51,52,53]. However, in the same cells, PDK1 can directly activate S6K1 and S6 protein by-passing AKT phosphorylation [51,52,53]. We cannot exclude a similar mechanism functioning in human keratinocytes since PI3Kδ sustains the phosphorylation of PDK1 and S6 protein induced by IL-22 with similar induction kinetics. In addition, PI3Kδ also contributes to PDK1 and S6 phosphorylation in resting keratinocytes, and this effect seems to be independent on AKT phosphorylation, as shown in Figure 2.

Interestingly, PI3Kδ inhibition in psoriatic keratinocytes also interfered with STAT3 activation, the principal mediator of IL-22 signaling [41], known to be upregulated in psoriatic epidermis [42,54]. Of interest, it has been previously demonstrated that the existence of crosstalk between PI3K and STAT3 signaling in human cancer PI3K transformed cells [43], where the pharmacological inhibition of PI3K prevented Tyr705 phosphorylation of STAT3. Important, in these cells, STAT3 activation by PI3K was mediated by a member of the Tec kinases family since a specific Tec kinase inhibitor impaired STAT3 phosphorylation and interfered with PI3K-induced oncogenic transformation. In addition, the mTOR inhibitor rapamycin did not counteract STAT3 phosphorylation, reinforcing the role of Tec kinases as a link between PI3K and STAT3 upstream of the mTOR kinase [43].

STAT3 controls inflammation and de-differentiative programs induced by IL-22 in human keratinocytes [42,45]. In line with this assessment, PI3Kδ inhibition by seletalisib reduces the expression of inflammatory chemokines such as *CXCL8* and *CXCL1* and restores the levels of the differentiation markers K10 and loricrin impaired by IL-22, thus mimicking the effects of STAT3 silencing observed by Sestito et al. in human keratinocytes [42]. Therefore, we propose that the pro-differentiative effects executed by PI3Kδ inhibition could be related not only to PI3K/AKT downregulation, as demonstrated in differentiated epidermal keratinocytes [30], but also to STAT3 inactivation (Figure 8).

Epidermis homeostasis in healthy skin is finely regulated not only by the balance between proliferation and differentiation of keratinocytes but also by cell death programs that are tightly controlled to ensure a proper cutaneous thickness and epidermal barrier function. Keratinocytes of psoriatic skin are characterized by a peculiar resistance to cytokine-induced apoptosis, thus contributing to the epidermal structural alterations [48,55]. Hyperactivation of AKT has been demonstrated to prevent cytokine-induced apoptosis via NF-κB-p65 pathway [7]. In particular, NF-*κ*B pathway protects human keratinocytes from apoptosis by inducing the expression of anti-apoptotic proteins, such as BCL-2, and in parallel, by phosphorylating and inactivating the pro-apoptotic BAD molecule, thus leading to the suppression of pro-apoptotic mechanisms [56]. In support of this, the chemical inhibition of PI3K/AKT by Ly294002 renders psoriatic keratinocytes more susceptible to pro-apoptotic stimuli, such as IFN-γ and TNF-α [7]. Coherently, we demonstrated that PI3Kδ inhibition by seletalisib renders psoriatic keratinocytes more susceptible to TNF-*α*-induced apoptosis (Figure 8). Considering that we did not observe any difference in PI3Kδ protein levels between psoriatic and healthy keratinocytes, we can explain apoptosis results by supposing a more sustained activation of PI3Kδ by inflammatory cytokines in psoriatic cells, compared to healthy strains (data not shown).

Upon TNF-α exposure, PI3K/AKT pathway also induces immune and inflammatory responses through p65 phosphorylation [57]. In accordance with this, the selective inhibition of PI3Kδ resulted in the reduced expression of several inflammatory mediators and, as proposed in Figure 8, these effects can be mechanistically explained with the strong inhibition of PDK1 and, consequently, of AKT and p65 phosphorylation.

In this study, the proliferative and inflammatory action of PI3Kδ in psoriasis context has been confirmed in the in vivo murine model of psoriasiform dermatitis induced da IMQ. Here, PI3Kδ is strongly upregulated in infiltrating immune populations and in keratinocytes of spinous and basal epidermal layers, thus reflecting the expression pattern observed in psoriatic skin lesions. In contrast to AKT phosphorylated in Ser473, whose expression is confined to suprabasal keratinocytes, the expression of AKT phosphorylated in Thr308 correlates to that of PI3Kδ and Ki67, all observed in keratinocytes of basal and spinous epidermal layers. PDK1 is also hyperactivated in IMQ-psoriasiform skin lesions, thus suggesting a relevant role for PI3Kδ/PDK1/p-AKT Thr308 axis in epidermal hyperplasia of IMQ-psoriasis like model.

Topical administration of seletalisib significantly attenuates the severity of psoriasiform phenotype induced by IMQ, by reducing the epidermal thickness in association with the decrease of the expression of markers of proliferation, and by restoring the physiological proliferation and differentiation programs in keratinocytes. Moreover, PI3Kδ inhibition resulted in a reduced infiltration of neutrophils, which is associated with the decrease of neutrophilic chemoattractants (i.e., *Cxcl15*), as well as of T CD3^+^ lymphocytes. Of note, PI3Kδ inactivation by seletalisib resulted in a strong decrease of *Il-17a* and *Il-22* cytokines that are mainly produced by γδT cells in IMQ model [14,58,59]. Consistently, the expression of *Il-1β* and *Ccl20*, responsible for the proliferation and epithelial recruitment of γδT cells, respectively [60], was inhibited by seletalisib. Additionally, *Tnf-α* and *Il-36γ*, strongly released by epidermal keratinocytes following TLR7/8 activation in IMQ model [36,61,62], were reduced by seletalisb.

Thus, we can propose that the anti-proliferative and anti-inflammatory effects determined by PI3Kδ inhibition are associated to the impairment of PDK1/p-AKT (Thr308) activation, whereas the restoration of terminal differentiation could be related to the reduction of p-AKT Ser473 in suprabasal layers of mice epidermis. It is worth mentioning that seletalisib also determined a decrease of PI3Kδ expression in both infiltrating immune cells and basal keratinocytes, suggesting a feedback regulation, likely also due to the reduction of TNF-α and IL-22, the main cytokine triggers of PI3Kδ expression.

Finally, administration of MK2206 inhibitor, inhibiting the downstream AKT molecule, resulted less efficacious in the amelioration of psoriasis-related symptoms in IMQ model. This observation supports the hypothesis that PI3Kδ sustains AKT-independent molecular pathways such as PI3Kδ/PDK1/S6 or PI3Kδ/STAT3 axis (Figure 8). A minor ameliorative impact was also observed with Ly294002, a pharmacological inhibitor of all PI3K isoforms, likely due to its lower biochemical affinity to PI3K targets compared to seletalisib. These in vivo results were in line with our preliminary in vitro data, demonstrating the reduction of the transcriptional expression of a limited number of inflammatory genes in TNF-α-activated keratinocytes treated by MK2206 or Ly294002.

In conclusion, we propose for the first time a role for PI3Kδ in the regulation of pathological processes executed by keratinocytes in psoriasis. Transcriptomic analysis of human keratinocytes silenced for PI3Kδ will identify possible molecular links and biological programs mediated by PI3Kδ, not yet unveiled.

Finally, despite of the availability and efficacy of systemic treatments targeting inflammatory cytokines in psoriasis management, we suggest that PI3Kδ inhibition could be effective in topical treatment of psoriatic lesions not only by contrasting epithelial inflammation but also by interfering with the epidermal aberrations of diseased skin. However, due to the importance of PI3K/AKT signaling in cancer [63,64,65], PI3Kδ could represent a highly attractive drug target also for the treatment of skin tumors and, in particular, of non-melanoma skin cancers, characterized by hyperproliferation of epidermal keratinocytes.

## Figures and Tables

**Table 1:** List of primer sequences used for real-time PCR analysis on human and mouse samples.

**Human**		**Sequence**
*CXCL8*	Fw	5′-CCC CTA AGA GCA GTA ACA GTT CCT-3′
	Rv	5′-GGT GAA GAT AAG CCA GCC AATC-3′
*CCL2*	Fw	5′-CAC CAG CAG CAA GTG TCCC-3′
	Rv	5′-CCA TGG AAT CCT GAA CCC AC-3′
*HBD-2*	Fw	5′-TCC TCT TCT CGT TCC TCT TCA TATT-3′
	Rv	5′-TTA AGG CAG GTA ACA GGA TCGC-3′
*HBD-2 probe*	Fw	5′-ACC ACC AAA AAC ACC TGG AAG AGG CA-3′
*GM-CSF*	Fw	5′-GCG TCT CCT GAA CCT GAG TAG-3′
	Rv	5′-TCG GCT CCT GGA GGT CAA AC-3′
*CCL5*	Fw	5′-CTACTGCCCTCTGCGCTCC-3′
	Rv	5′-TGGTGTCCGAGGAATATGGG-3′
*CXCL1*	Fw	5′-CCTCAATCCTGCATCCC-3′
	Rv	5′-AGTTGGATTTGTCACTGT-3′
*HPRT1*	Fw	5′-TGACACTGGCAAAACAATGCA-3′
	Rv	5′-GGTCCTTTTCACCAGCAAGCT′-3′
**Mouse**		**Sequence**
*Ccl20*	Fw	5′-AGG CAG AAG CAG CAA GCA AC-3′
	Rv	5′-ACA AGC TTC ATC GGC CAT CT-3′
*Il-36γ*	Fw	5′-AGCAGGTGTGGSTCTTTCGT-3′
	Rv	5′-ACGCTGACTGGGGTTACTCT-3′
*S100a7*	Fw	5′-AGC AAC AGA CTC TCC GCT G-3′
	Rv	5′-CTG GCA TGA CTG ATG GAC CC-3′
*Il-22*	Fw	5′-TGT TCC GAG GAG TCA GTG CTA-3′
	Rv	5′-CAG AAC GTC TTC CAG GGT GA-3′
*Tnf-α*	Fw	5′-GTC CCC AAA GGG ATG AGA AGT T-3′
	Rv	5′-GGG TCT GGG CCA TAG AAC TG-3′
*Il-17a*	Fw	5′-AGA AGG CCC TCA GAC TAC CT-3′
	Rv	5′-CTT CAT TGC GGT GGA GAG TC-3′
*Hprt1*	Fw	5′-TCC TCA GAC CGC TTT TTG CC-3′
	Rv	5′-ATC GCT AAT CAC GAC GCT GG-3′

**Table 2:** Effects of Ly294002, MK2206, and seletalisib on the expression of inflammatory molecules in IMQ-psoriasis-like mouse model on the expression of pro-inflammatory molecules psoriasis-related.

Inflammatory Molecules	IMQ(−)	IMQ(+)	IMQ(+) w Ly294002	IMQ(+) w MK2206	IMQ(+) w Seletalisib
*Cxcl15*	5.21 ± 1.8	232.25 ± 52.3	9.42 ± 3.45 **	2.16 ± 0.8 **	1.00 ± 0.14 **
*Ccl20*	1.00 ± 0.12	7859.84 ± 78.22	3311.33 ± 43.56 *	8450.58 ± 143.22	3325.83 ± 54.12 **
*Il-1β*	1.00 ± 0.02	161.37 ± 43.23	171.18 ± 38.92	177.99 ± 41.65	57.34 ± 18.76 *
*Il-17a*	1.00 ± 0.18	465.80 ± 56.87	552.09 ± 48.82	492.95 ± 35.98	215.65 ± 40.53 *
*Tnf-α*	1.00 ± 0.08	43.83 ± 12.43	50.96 ± 9.87	49.33 ± 12.32	17.32 ± 8.98 *
*Il-22*	1.00 ± 0.14	513.02 ± 29.81	83.60 ± 12.56 *	578.00 ± 39.45	84.12 ± 30.45 *
*S100a7*	1.00 ± 0.09	2384.84 ± 98.65	2193.30 ± 87.45	2065.05 ± 79.65	609.66 ± 35.56 **
*Il-36γ*	1.00 ± 0.03	11.17 ± 1.21	11.41 ± 1.80	12.49 ± 2.01	7.51 ± 1.98 *

Data reported in table show mean values ± SD obtained from real-time PCR analyses of *Cxcl15*, *Ccl20*, *Il-1β*, *Il-17a*, *Tnf-α*, *Il-22*, *S100a7*, and *Il-36γ* performed on pooled mRNA samples of dorsal skin biopsies of mice treated as indicated (*n* = 2 mice, Control IMQ (−); *n* = 6 mice, IMQ (+); *n* = 6 mice, IMQ (+) w Ly294002; *n* = 6 mice, IMQ (+) w MK2206; IMQ (+) w seletalisib groups) and normalized to *Hprt1* levels. * *p* ≤ 0.05, ** *p* ≤ 0.01, as assessed by unpaired Student‘s *t* test.

## Data Availability

Primary data: data access: GSE13355 and GSE41662.

## Supplementary Materials

The following are available online at https://www.mdpi.com/article/10.3390/cells10102636/s1, Figure S1: Seletalisib treatment does not induce cytotoxic effect on psoriatic keratinocytes but downregulates activation of PI3K effectors in a dose-dependent manner. Figure S2: PI3Kδ inhibition reduces the expression of inflammatory genes induced by IL22-activated psoriatic keratinocytes. Figure S3: PI3Kδ inhibition does not alter the apoptotic rate of TNF-α-activated healthy keratinocytes. Figure S4: Seletalisib has ameliorative in vivo effects broader than those observed with Ly294002 or MK2206 in IMQ model. Figure S5: Seletalisib administration interferes with PI3Kδ-related signaling pathways in IMQ-induced psoriasiform model.
