# Supplementary material for: PI3Kδ Sustains Keratinocyte Hyperproliferation and Epithelial Inflammation: Implications for a Topically Druggable Target in Psoriasis
Source: Cells. 2021 Oct 2;10(10):2636. doi: 10.3390/cells10102636 (PMC8534452; doi:10.3390/cells10102636)
Supplement: Supplementary file 1 [file cells-10-02636-s001.zip › cells-1390868-supplementary.pdf]

A

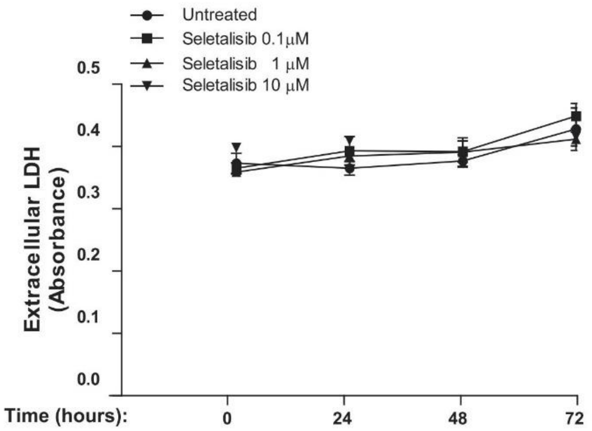

B

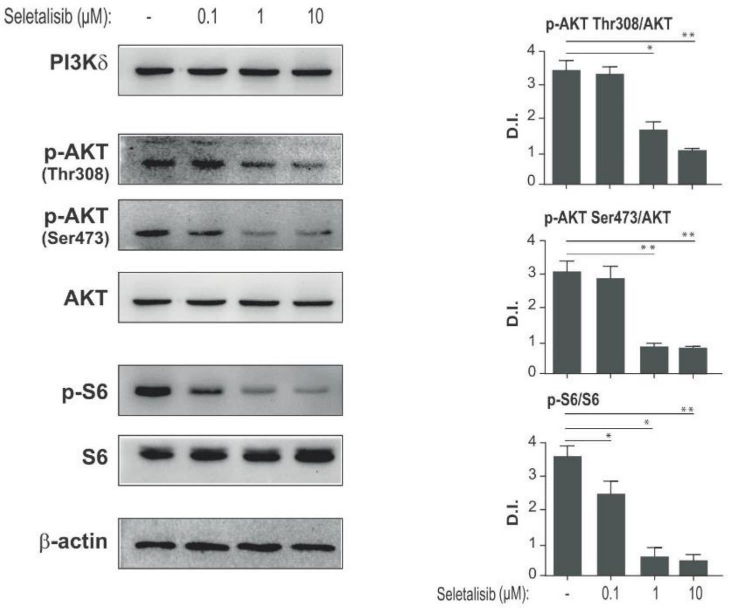

Supplementary Figure S1. Seletalisib treatment does not induce cytotoxic effect on psoriatic keratinocytes but downregulates activation of PI3K effectors in a dose-dependent manner. **(A)** Cytotoxicity towards different doses of seletalisib was tested by measuring LDH extracellular activity from supernatants of psoriatic keratinocyte cultures. Cells were left untreated or exposed to different doses of seletalisib (0.1-1-10 µM) for 24, 48 and 72 hours as indicated. Data are shown as mean values of absorbance [492 nm-690 nm] obtained from three independent experiments ± SD. **(B)** Protein extracts were obtained from psoriatic keratinocytes in presence or absence of different doses of seletalisib (0.1-1-10 µM) for 6 hours, and subjected to WB analysis to detect PI3Kδ expression, and phosphorylation of AKT (Thr308, Ser473) and S6 proteins. Filters were re-probed with anti-AKT and -S6. β-actin levels were detected as a loading control. One representative experiment out of three performed is shown. Graphs shown reported D.I. of bands obtained from WB analysis of protein extracts obtained from psoriatic keratinocytes (*n* = 3 strains). Data are expressed as mean ± SD of each D.I. value of total protein levels or the ratio of phosphorylated/unphosphorylated proteins normalized to

β-actin expression. Results were obtained by three independent experiments. *p ≤ 0.05, **p ≤ 0.01 were assessed by using a paired Student's t test comparing seletalisib-treated cells with untreated cells.

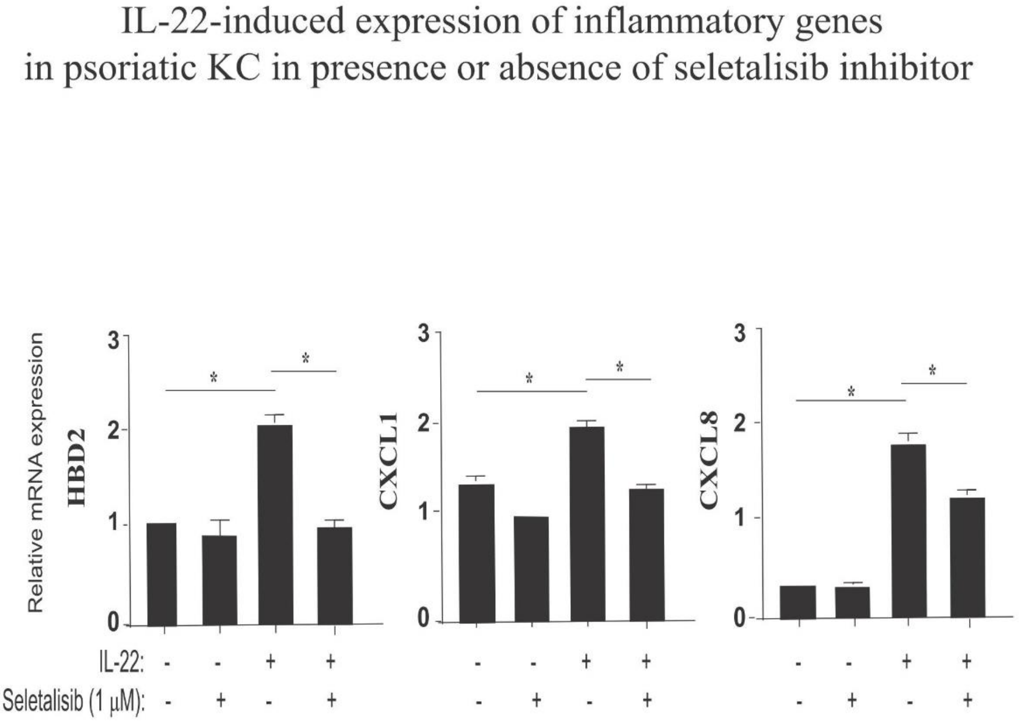

Supplementary Figure S2. PI3Kδ inhibition reduces the expression of inflammatory genes induced by IL22-activated psoriatic keratinocytes. Keratinocyte cultures were left untreated or stimulated with IL-22 in presence or absence of seletalisib for 8 hours, *HBD2*, *CXCL1*, *CXCL8* mRNA expression was detected by real-time PCR and normalized to HPRT1 levels. Data shown are the mean of three different experiments ± SD. *p ≤ 0.05, as assessed by paired Student’s t test.

Evaluation of TNF-α-induced apoptosis in healthy KC in presence or absence of seletalisib inhibitor

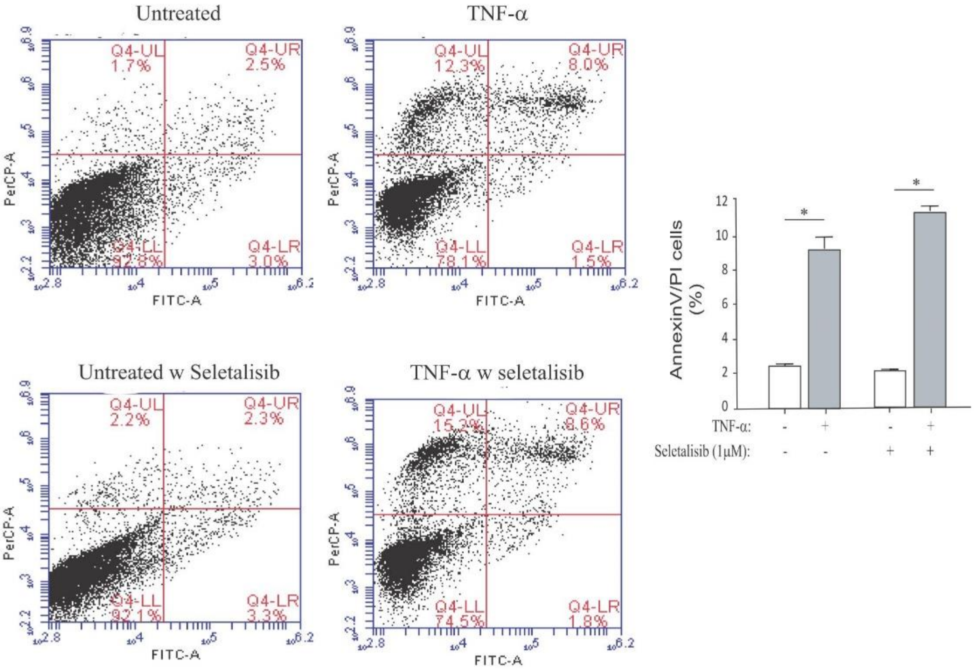

Supplementary Figure S3. PI3Kδ inhibition does not alter the apoptotic rate of TNF-α-activated healthy keratinocytes. Healthy keratinocytes were pre-treated or not with seletalisib (1 μM) and then stimulated with TNF-α for 48 hours. Apoptosis was evaluated by measuring Annexin/PI fluorescent staining through FACS analysis. Graphs show the mean ± SD of the percentage of AnnV/PI double positive cells of three independent experiments. *$p \leq 0.05$, as calculated by paired Student's t test.

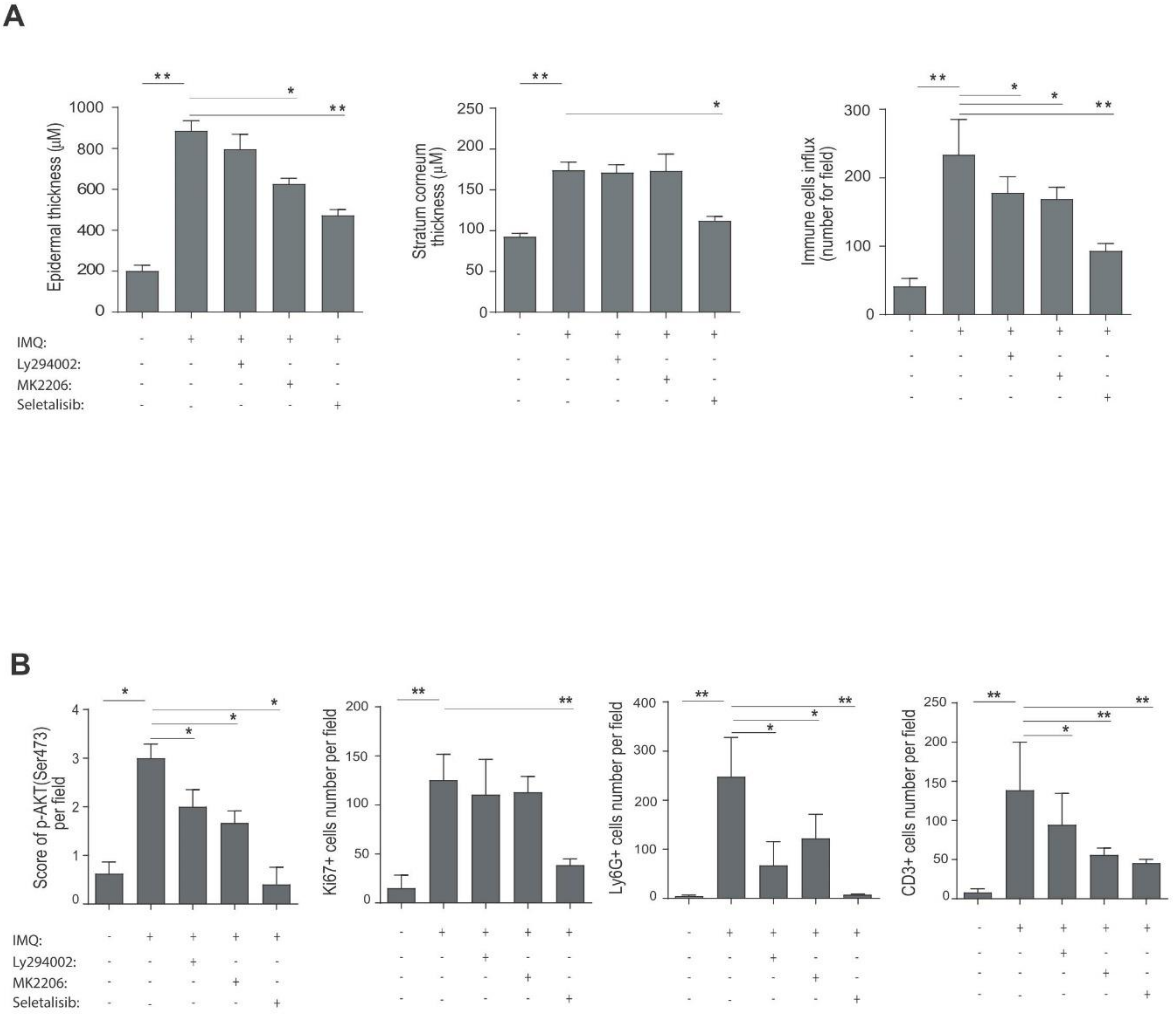

Supplementary Figure S4. Seletalisib has ameliorative *in vivo* effects broader than those observed with Ly294002 or MK2206 in IMQ model. **(A)** Quantifications of epidermal and stratum corneum thickness, and immune cell influx, were analyzed as parameters of skin acanthosis and inflammation. Measurements were obtained from H&E staining on mice treated as indicated. Graphs show means of microns of epidermis and stratum corneum thickness, and mean of number of infiltrating immune cells per section ($n = 3$ sections) ± S.D. per group ($n = 2$ mice, Control IMQ(-); n = 6 mice, IMQ(+); n = 6 mice, IMQ(+)/Ly294002; n = 6 mice, IMQ(+)/MK2206; n = 6, IMQ(+)/seletalisib groups). p* ≤ 0.05 and **p ≤ 0.01, as assessed by unpaired Student's t test. **(B)** Quantifications of pathological markers obtained from IHC analysis of skin sample sections from control IMQ(-), IMQ(+), IMQ(+) w Ly294002, IMQ(+) w MK2206 and IMQ(+) w seletalisib groups. Graphs shown represents the mean of four-stage score values for p-AKT (Ser473) expression, or the mean of number of positive cells for Ki67, Ly6G, and CD3 expression analysis, per three sections per all mice of each experimental group. *p ≤ 0.05, **p ≤ 0.01, as assessed by unpaired Student's t test.

Expression profile of PI3Kδ effectors in murine skin of IMQ-induced psoriasiform model topically treated or not with seletalisib

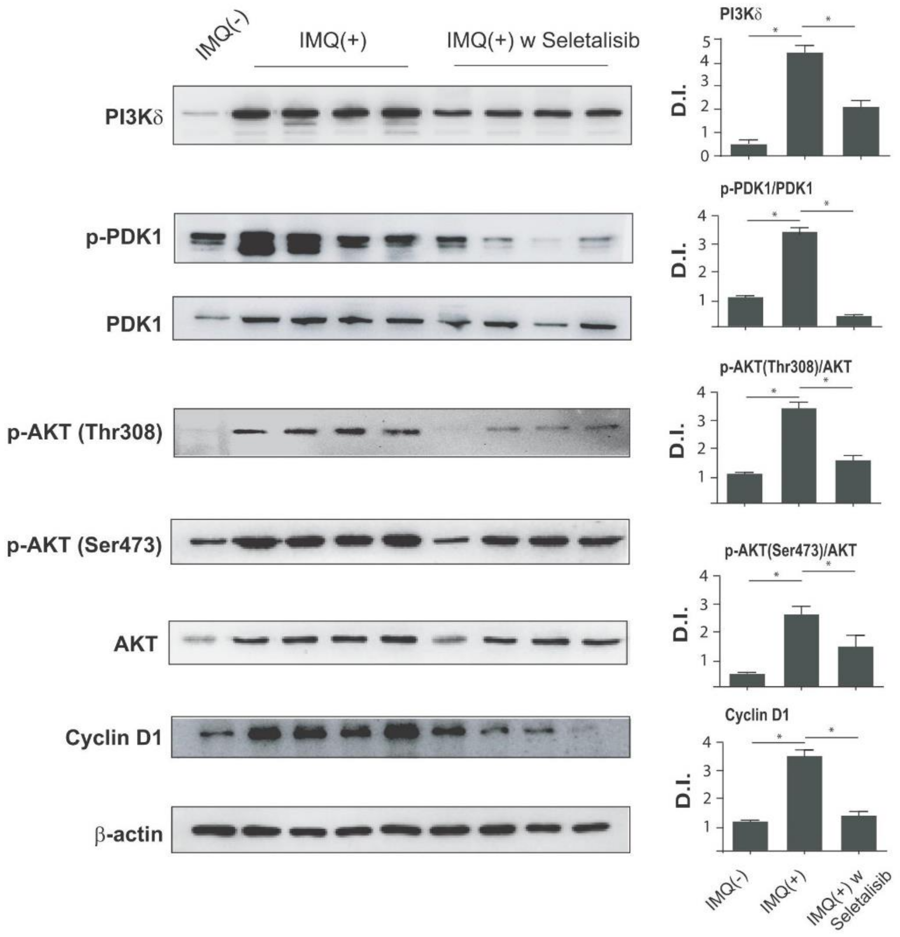

Supplementary Figure S5. Seletalisib administration interferes with PI3Kδ-related signaling pathways in IMQ-induced psoriasiform model. Protein extracts were obtained from skin samples of IMQ(-), IMQ(+) and IMQ(+)/seletalisib mice groups and subjected to WB analysis to detect PI3K phosphorylated-AKT (Thr308; Ser473), phosphorylated-PDK1 and cyclin D1. Filters were re-probed with anti-PDK1 and -AKT antibodies. β-actin levels were detected as a loading control. One representative WB experiment out of three performed is shown, four mice from IMQ(+) and four from IMQ(+)/Seletalisib groups were compared to one mice from IMQ(-) mice group. Graphs reports the mean values of D.I. of bands obtained from all animals of each group. D.I. values refer to the total protein levels or the ratio of phosphorylated/unphosphorylated proteins, all normalized to β-actin expression. $*p \leq 0.05$, $**p \leq 0.01$, as assessed by paired Student's t test.
